# A SNARE Protein Identification Method Based on iLearnPlus to Efficiently Solve the Data Imbalance Problem

**DOI:** 10.3389/fgene.2021.818841

**Published:** 2022-01-28

**Authors:** Dong Ma, Zhihua Chen, Zhanpeng He, Xueqin Huang

**Affiliations:** Institute of Computing Science and Technology, Guangzhou University, Guangdong, China

**Keywords:** SNARE protein identification, ASDC features, SMOTE, data imbalance, machine learning

## Abstract

Machine learning has been widely used to solve complex problems in engineering applications and scientific fields, and many machine learning-based methods have achieved good results in different fields. SNAREs are key elements of membrane fusion and required for the fusion process of stable intermediates. They are also associated with the formation of some psychiatric disorders. This study processes the original sequence data with the synthetic minority oversampling technique (SMOTE) to solve the problem of data imbalance and produces the most suitable machine learning model with the iLearnPlus platform for the identification of SNARE proteins. Ultimately, a sensitivity of 66.67%, specificity of 93.63%, accuracy of 91.33%, and MCC of 0.528 were obtained in the cross-validation dataset, and a sensitivity of 66.67%, specificity of 93.63%, accuracy of 91.33%, and MCC of 0.528 were obtained in the independent dataset (the adaptive skip dipeptide composition descriptor was used for feature extraction, and LightGBM with proper parameters was used as the classifier). These results demonstrate that this combination can perform well in the classification of SNARE proteins and is superior to other methods.

## Introduction

Soluble N-ethylmaleimide-sensitive fusion protein attachment protein receptor (SNARE) proteins are a small superfamily of proteins. They have an uncomplicated domain structure, and a feature of them is the SNARE motif—an evolutionarily conserved heptanucleotide repeat consisting of 60–70 amino acids. (Jahn and Scheller, 2006) They can be divided into Q-SNAREs and R-SNAREs pursuant to the structural characteristics of SNAREs. Functionally, SNAREs are most likely associated with various aspects of membrane transport specificity, and they are a key element in membrane fusion and are necessary for stable fusion intermediates. (Schoch et al., 2001) SNARE proteins are involved in membrane vesicle transport, such as synaptic transmission between nerve cells (synaptic vesicle transport) and plant disease resistance (disease resistance signaling). In addition, SNAREs are also implicated in the formation of some mental disorders. (Wang et al., 2018)

It is relatively complex to explore the function of a particular protein in the field of biology, the general prediction method is based on Protein-Protein-Interaction (PPI) (Hu et al., 2011; Zhai et al., 2020; Sundar and Narmadha, 2021) and protein structure information (Kinjo and Nakamura, 2012; Sharma and Srivastava, 2021). In the subsequent process, the specific function of detection through the complex biological experiment needs to be clear, which greatly increases the difficulty and the resources required of the properties that determine protein function, thus reducing the efficiency due to unavoidable time consumption.

In recent years, with the development of machine learning, many methods have achieved good results in various fields, such as Nature Language Processing (NLP) and computer vision (Jin et al., 2021). In addition, the classification task is one of the most basic applications in machine learning, and relevant research is has matured. (Ke et al., 2017) Nguyen Quoc Khanh Le, et al. (Le et al., 2019) employed PSSM profiles and 2D CNN to identify SNARE proteins. Su, Xin, et al. (Su et al., 2019) applied the multiscale convolutional network to the identification of antimicrobial peptides, so it is appropriate to apply machine learning to protein classification tasks.

In this paper, multiple feature extraction algorithms are used to extract different features, obtain the best performance descriptor through performance comparison, and then perform data enhancement processing on the extracted features of this descriptor to address the problem of sample imbalance in the data to a certain extent. Finally, the processed feature data and raw data of the independent test set were used to train the classifier to obtain the eventual model.

## Materials and Methods

**FIGURE 1:**
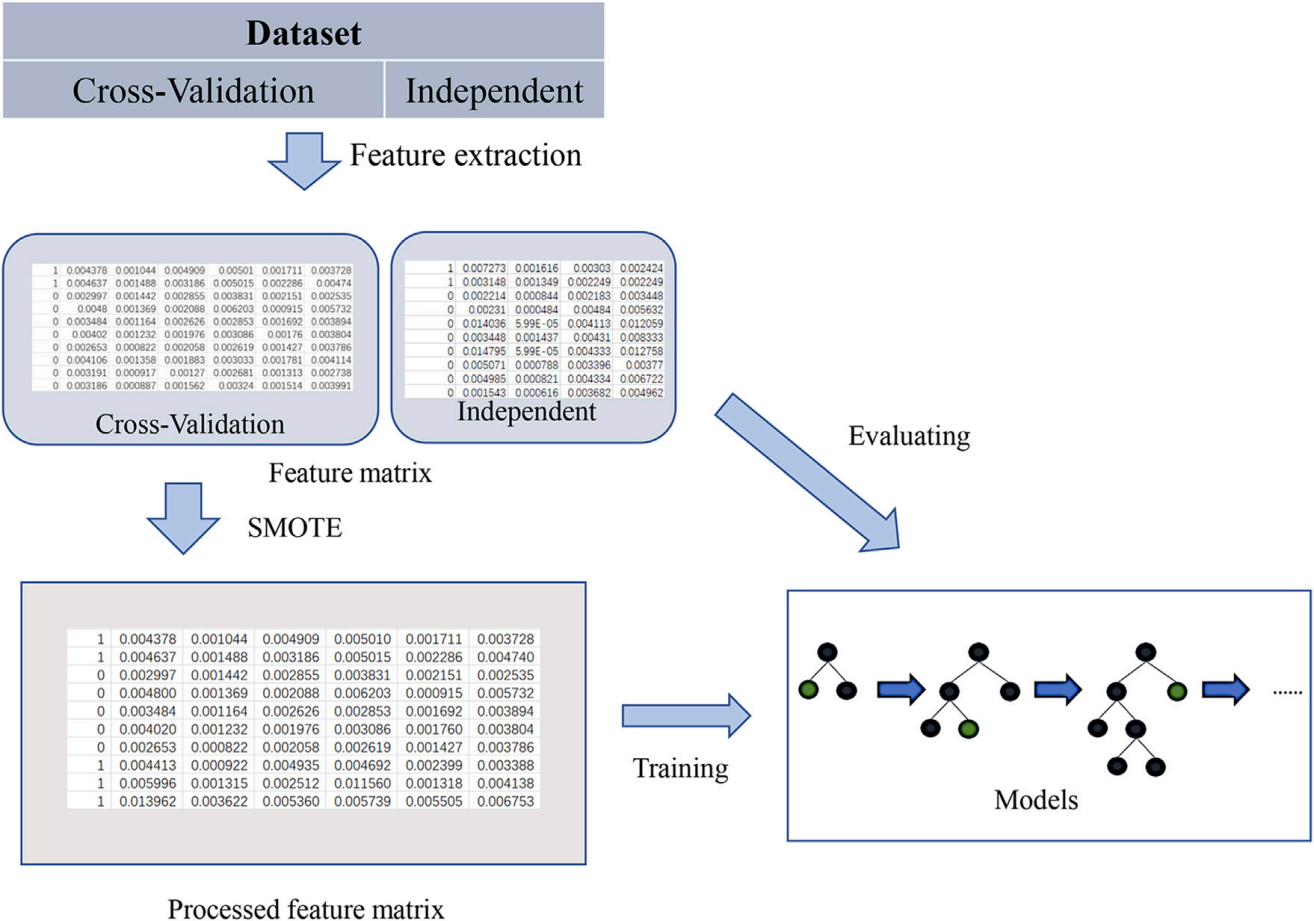
The research flow diagram of SNARE protein identification using a decision tree model.

The task of protein sequence classification models based on machine learning generally includes five main steps: protein sequence data collection, feature extraction and processing, classifier construction and optimization, model performance evaluation, and result visualization. (Liu et al., 2019; Guo et al., 2020; Tao et al., 2020; Chen Z et al., 2021; Li et al., 2021) The details of the first three steps determine whether the classification performance is satisfactory, while the last two steps are only a further explanation of the experimental results and determined by objective evaluation indicators, so the sequence classification task is mainly carried out using the first three steps. Figure 1 illustrates the research flow of this paper.

### Datasets

The research object of this paper are SNARE proteins, which are generally downloaded from the UniProt database. As the research object is a specific type of protein, less sequence data can be obtained for a specific protein compared to other non-specific types of common proteins, which leads to the final dataset being easily unbalanced, i.e., the number of nonspecific proteins in the dataset is greater than the number of specific proteins. The dataset used in this study was from other similar tasks. (Le and Nguyen, 2019) The number of SNARE proteins in this dataset was only one-tenth that of non-SNARE proteins, including 697 SNARE proteins as positive samples and 7,378 vesicle transport proteins as negative samples. During the experiment, 90% of them were extracted for the training of the model, and the rest were used as independent validation sets to evaluate the generalization ability of the model.

### Feature Extraction and Processing

Biological sequence data are generally stored in a FASTA file format, and each sequence data is represented by the letter of the nucleotide or amino acid constituting the molecule. As the number of molecules composing the biological sequence is not fixed, the length of the sequence is inconsistent. However, traditional machine learning models can only deal with fixed-dimension data in digital format, so it is necessary to encode source sequence data into restricted-length digital data to meet the input requirements of the model, which is the feature extraction of sequential data. Descriptors are used in the first step of biological sequence analysis. They extract various biological sequence features from multiple perspectives, such as amino acid composition, biochemical characteristics, and residue composition, with different emphases and features. Consequently, these algorithms may have different performances for various sequence analysis tasks. Typically, the most appropriate algorithm for a given task needs to be obtained by testing various feature extraction algorithms on the dataset and comparing the performance of each algorithm.

### Treatment of Data Imbalance

As mentioned above, the number of positive samples in the dataset used in this paper is only one 10th of the number of negative samples, which will lead to unbalanced recognition of positive and negative samples in training process and affect the final classification results (Zou et al., 2016; Cheng et al., 2018; Azad et al., 2019; Priya and Sivaraj, 2021; Shao et al., 2021). The model trained with unbalanced data will be more inclined to fit the negative instances with a large number, which will lead to the degradation of the model’s classification performance for the small number of positive samples. Since there are more negative samples in the dataset than positive samples, if the source files are directly used for training, the classifier will learn too many negative samples, thus reduce the recognition ability of the model for positive samples, but this is contrary to our main purpose. Therefore, it is necessary to adopt some strategies to alleviate the problem of sample imbalance. The relatively small number of specific proteins in nature and the widespread sample imbalance in the field of biological sequence classification had also led to abundant research on the processing of unbalanced data. (Chao et al., 2019; Kaur et al., 2019; Yang et al., 2020; Ao et al., 2021a; Shao and Liu, 2021) The most common are oversampling and downsampling. Oversampling is balanced by adding redundant samples to a small number of positive samples, and the strategy can improve the recognition ability of positive samples to a certain extent, but it simply repeats positive examples and overemphasizes existing positive examples, which would urge the risk of overfitting positive examples. In the downsampling method, only a portion of the negative samples is selected for lower sampling to reduce the number of negative samples. However, this method can only improve the model’s classification ability of positive samples to a certain extent. Because a few of the counterexample data are discarded, their influence in the overall sample is reduced, which may result in a large deviation model, and greatly affect the overall performance.

Considering the serious imbalance between positive and negative samples in this dataset, only one unbalanced strategy may not work well; it needs to be sampled up and down simultaneously. This article uses a combination of sampling partial negative samples and the synthetic minority oversampling technique (SMOTE) to generate new positive samples to address sample imbalance. (Chawla et al., 2002; Riaz and Li, 2019; Zhang C H et al., 2020; Zhao et al., 2020)

SMOTE is an oversampling technique that balances the quantity gap between two categories by finding the nearest neighbor of certain data in a positive example and then using the K-nearest neighbor algorithm to generate new positive samples. For each sample *x* in the positive sample, calculate the K positive samples *x*_*k*_ {k = 1, 2, K} closest to *x*, and determine the sampling ratio n according to the unbalanced proportion of samples. For the k nearest neighbor samples of each sample *x*, n samples are randomly selected, and the newly constructed sample *x*_*new*_ can be obtained through the following formula:

$$x_{new}=x+rand\left(0,1\right)*\left|x-x_{n}\right|$$ (1)

In the experiment, part of the negative sample is treated with simple undersampling at first. SMOTE is used to generate positive samples to ensure that the number of positive and negative samples is consistent. Then, a balanced dataset of sample size can be obtained, which will be used in subsequent model training experiments.

## Result and Discussion

### Evaluation Indexes

To objectively evaluate the performance of various algorithms, some convincing indicators of these algorithms need to be compared after the experiment (Wei et al., 2017; Wei et al., 2018; Wei et al., 2019; Wang et al., 2020; Ding et al., 2021; Shang et al., 2021; Wu and Yu, 2021; Yang et al., 2021). Next, the algorithm with the best performance is selected for subsequent research according to these indices. Similarly, common metrics are used to compare the performance of each algorithm. The four values of TP, FP, TN, and FN (representing true positive, false-positive, true negative, and false negative values, respectively) can be obtained for the classifier test (Jiang et al., 2013; Cheng et al., 2016; Xiao et al., 2019; Zhang L et al., 2020; Huang et al., 2020; Li and Liu, 2020; Liu et al., 2020; Mo et al., 2020; Tang et al., 2020; Han et al., 2021; Wang et al., 2021; Xu et al., 2021). Accuracy, MCC, sensitivity, and specificity can then be calculated based on these values.

$$Sensitivity=\frac{TP}{TP+FN}$$ (2)

$$Specificity=\frac{TN}{TN+FP}$$ (3)

$$Accuracy=\frac{TP+TN}{TP+FP+TN+FN}$$ (4)

$$MCC=\frac{TP\times TN-FP\times FN}{\sqrt{\left(TP+FP\right)\left(TP+FN\right)\left(TN+FP\right)\left(TN+FN\right)}}$$ (5)

### Selection of the Descriptors

In this paper, the iLearnPlus platform (Chen Z et al., 2021) was used to compare the performance of various extraction algorithms: multiple descriptors were applied to obtain the feature vectors of the source FASTA file, followed by training and testing the obtained features using several classification algorithms and analyzing the performance of different feature extraction algorithms. To eliminate the influence of other subjective factors, the area under the receiver operating characteristic curve (AUROC) index was adopted to evaluate the performance of the algorithm.

Accuracy and MCC are widely adopted to measure model performance in classification problems. These two values can be regulated by artificially setting thresholds so that the specific performance of each algorithm cannot be truly reflected. The AUROC index takes TPR [TP/(TP + FN)] and FPR[FP/(FP + TN)] as the horizontal and vertical coordinates to obtain the area under the curve. The larger the area is, the higher the coincidence degree between the prediction label of the model and the source label is. It is necessary to take the AUROC as the evaluation standard so that the algorithm with the best overall performance can be selected.

According to the experiment, several feature extraction algorithms and classifiers with better performance can be obtained. Some experimental results are shown in Table 1.

**TABLE 1:** Feature dimensions of partial feature extraction algorithms and AUROC performance under multiple classifiers.

	Feature dimension	RandomForest (Breiman, 2001)	LightGBM (Ke et al., 2017)	XGBoost (Chen and Guestrin, 2016)
ASDC (Wei et al., 2018)	400	**0.8599**	**0.8829**	**0.8839**
QSOrder (Chou, 2000)	44	0.8401	0.864	0.8604
DDE (Saravanan and Gautham, 2015)	400	0.824	0.8604	0.849
CKSAAP (Chen et al., 2007)	1,600	0.8337	0.8664	0.8588
AAC (Bhasin and Raghava, 2004)	20	0.8467	0.8514	0.8428

The meaning of the bold values is the feature extraction algorithm that performs best under a particular classification algorithm.

The experimental results show that the performance of adaptive skip dipeptide composition (ASDC), CKSAAP, and QSOrder feature extraction algorithms outperform other algorithms. Among them, the optimal algorithm is the ASDC, and the subsequent multiple numbers also use ASDC to extract features.

ASDC is a feature extraction algorithm based on GDC (G-gap dipeptide composition) algorithm. Dipeptide composition is the fraction of any two adjacent residues as a dipeptide pair, and it measures the correlation of any two adjacent residues in the peptide sequence. GDC encapsulates the composition and local order information of any two spacer residues in the peptide sequence, it has a hyperparameter *g* to determine the gap between two adjacent residues. And ASDC calculates all values of *g* and accumulates them. For a given protein read R with L length, the feature vector for ASDC is represented by:

$$ASDC=\left(fv_{1},fv_{2}...,fv_{400}\right)$$ (6)

where *fv*_*i*_ is calculated by

$$fv_{i}=\frac{\sum_{g=1}^{L-1}O_{i}^{g}}{\sum_{i=1}^{400}\sum_{g=1}^{L-1}O_{i}^{g}}$$ (7)

where *g* represents the g-gap (g = 1, 2, L-1) dipeptide and *fv*_*i*_ is the occurrence frequency of the ith (i = 1, 2, 400) adaptive skip dipeptide. It is worth mentioning that if the cumulative term with g is removed from Eq. 7, it becomes the formula for the GDC features.

Since there are approximately 8,000 samples in the dataset, the 400 dimension is relatively moderate. Another is that ASDC considers the frequency of any two unconnected amino acids in the whole protein and can capture all the information of dipeptide composition. It also shows that the SNARE proteins have a high correlation with their dipeptide composition. This information may bring biological assistance to the final SNARE protein recognition.

However, the results also showed that several other algorithms performed only slightly worse than ASDC, so it was considered that features stitched together after using multiple feature extraction algorithms could be used to train the model. After experimental verification, when the feature data extracted by algorithms such as ASDC and QSOrder were spliced together and then used to train the model, it was found that instead of improving the results, there was a slight decrease. In response to this result, it is believed that the data dimensionality is too large, and the resulting redundant data will not only have a positive effect on the training of the model but also degrade the model performance. Therefore, the spliced features were subsequently selected again, and relevant experiments were conducted. However, the model trained with these data still performed poorly on the independent set. After comparing the feature vectors extracted by the feature extraction algorithms used, it was concluded that the main reason was that the feature values obtained by each algorithm did not fall within the same range of values. For example, the feature matrix extracted by the QSOrder algorithm is a sparse matrix containing a large number of 0 or very close to 0 values, and there are some negative numbers in the DDE features, which when mixed together may affect the direction of the model iteration and thus the final results.

### Unbalanced Processing

In the step of dealing with the data imbalance problem, n negative samples are first downsampled from the original dataset to ensure that n is greater than the number of positive samples 628. Then, the SMOTE algorithm is used to expand the number of positive samples to n to build a balanced dataset. When *n* = 628, the strategy is equivalent to complete downsampling, and when *n* = 6,640 (the total number of negative samples), the strategy is equivalent to complete oversampling, so the value of n is in the range (628, 6,640). After sampling the negative samples, all data were tested with the same independent test set to determine their generalization ability.

To analyze the effect of the number of down samples n on the classification performance, several sets of parameters were set for experiments in this paper, and the best performing n value was selected based on the results. n values were set, and the related performance is shown in Table 2. To partially eliminate the error caused by the randomness of the data, no put-back sampling was performed in downsampling, and the set of negative samples sampled was denoted by S (n). Then, there was S(n)⊂S(m), where n < m.

**TABLE 2:** Model performance under different n values.

n	Cross-validation				Independent			
	Sens	Spec	Acc	MCC	Sens	Spec	Acc	MCC
628	82.97	82.954	82.486	0.6522	94.2	60.84	63.69	0.3102
1,256	95.148	89.886	92.516	0.8523	73.91	76.56	76.33	0.3152
2,510	98.486	91.832	95.158	0.9055	76.81	86.34	85.5	0.4492
3,764	99.07	94.394	96.73	0.936	65.22	93.22	90.83	0.5071
5,019	99.302	94.682	96.99	0.941	62.32	94.04	91.33	0.5081
6,640	99.292	94.414	96.852	0.9384	59.42	94.99	91.95	0.5149

### Parameter Optimization

In the recognition problem, it is also very important to select the appropriate classifier. There are also multiple classifiers in the field, each with a different focus, so their performance in a particular task may be different. Therefore, to select a classifier that best fits the task, we follow the same approach as in the selection of the descriptors subsection, where different classifiers are used to train and classify the same feature data, and the best performing algorithm is selected for subsequent experiments. After using three mainstream classifiers, the model performance corresponding to the parameters of Part n is shown in Tables 3 and 4. It can be concluded that LightGBM with n = 5,019 is the best performer and most in line with this task. LightGBM (Light Gradient Boosting Machine) is a framework for implementing the GBDT (Gradient Boosting Decision Tree) algorithm, which is an iterative decision tree algorithm consisting of multiple decision trees. LightGBM improves on the traditional GBDT algorithm in many ways, such as using a Histogram-based decision tree algorithm and using a leaf-wise strategy instead of level-wise.

**TABLE 3:** The performance of the three classifiers on the independent test set (n = 2,510).

n = 2,510	Sensitivity (%)	Specificity (%)	Accuracy (%)	MCC
RandomForest	63.77	**90.92**	**88.6**	0.444
LightGBM	**76.81**	86.31	85.5	**0.4492**
XGBoost	73.91	86.99	85.87	0.4412

The meaning of the bold values is the feature extraction algorithm that performs best under a particular classification algorithm.

**TABLE 4:** The performance of the three classifiers on the independent test set (n = 5,019).

n = 5,019	Sensitivity (%)	Specificity (%)	Accuracy (%)	MCC
RandomForest	46.38	95.66	91.45	0.435
LightGBM	**60.87**	**95.39**	**92.44**	**0.5386**
XGBoost	**60.87**	94.58	91.7	0.5132

The meaning of the bold values is the feature extraction algorithm that performs best under a particular classification algorithm.

**FIGURE 2:**
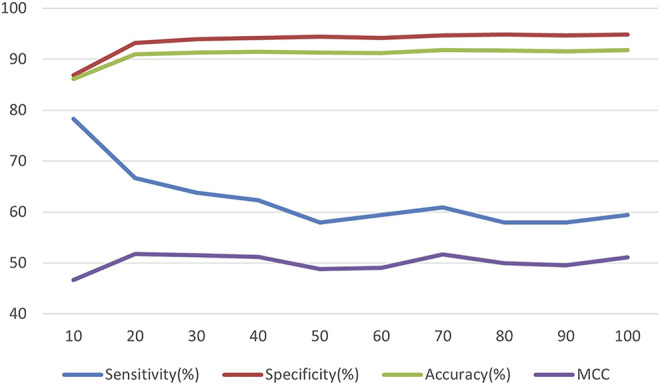
The relationship between the number of leaves and model performance.

In this experiment, the number of leaf nodes, the maximum depth of the tree and the learning rate of the LightGBM algorithm were adjusted (Ao et al., 2021b). First, we compared the impact of the number of leaf nodes of the tree on the performance of the algorithm when the maximum depth of the tree was not limited. The result is shown in Figure 2 (The MCC values in the figure have been normalized with the other three indicators for plotting purposes, and the following similar charts have been followed in the same way). Through a series of comparative experiments, the number of leaf nodes can be set to 31 while considering the efficiency of the algorithm operation.

**FIGURE 3:**
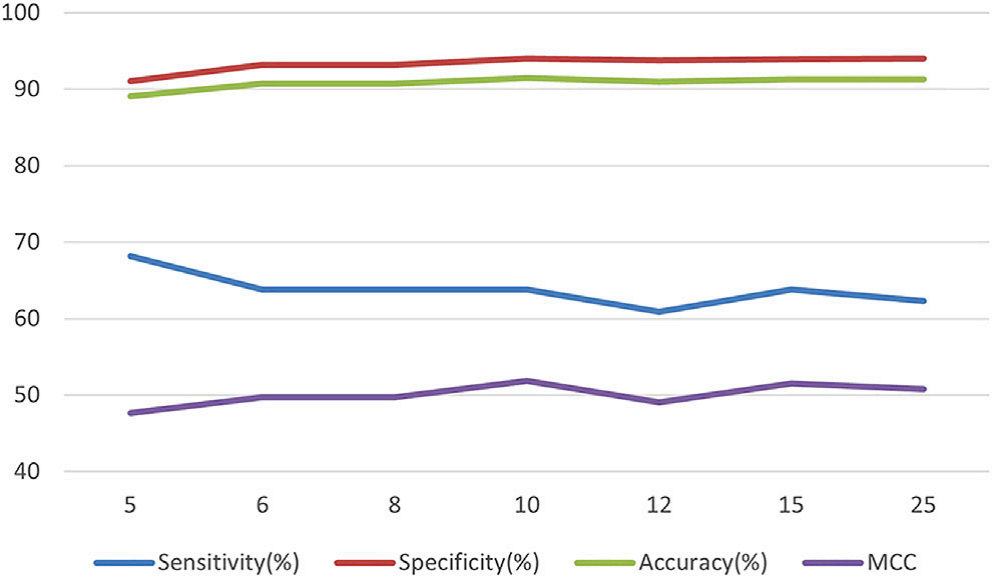
The relationship between the number of maxdepth and model performance.

This is followed by choosing the depth of the tree given the number of leaf nodes, as there is a maxdepth>2^leaves-1 constraint, and the leaf value has been set to 31; the maximum depth of the tree cannot be less than 5 (log2 (31 + 1). The result is shown in Figure 3. Similarly, the optimal maxdepth can be chosen as 10.

**FIGURE 4:**
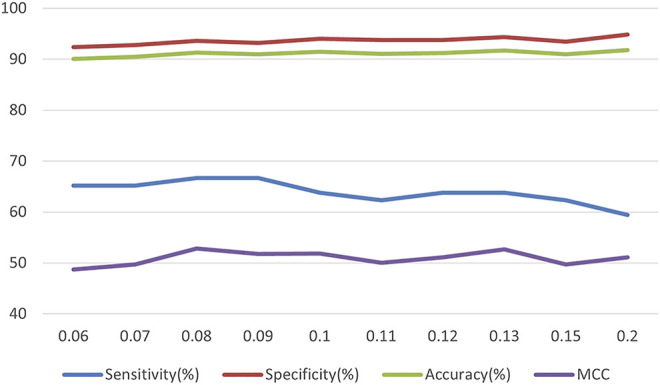
The relationship between learning rate and model performance.

Then, it is time to adjust the learning rate and compare the impact of changes in the learning rate on performance, and the results are shown in Figure 4. In the end, the optimal parameters are leaves = 31, maxdepth = 10, and learning rate = 0.08.

### Comparison With the Other Method

In comparison with 2D CNN, the data of this paper needed to be modified because the data allocation differed. It used a cross-validation set of 644 positive and 2,234 counterexamples and an independent dataset of 38 positive and 349 counterexamples. Similar experiments were conducted using this setup in this paper. In this sequence classification task, the focus is on the classification performance of the SNARE protein, which in the model performance evaluation is the size of the specificity. The experimental results are shown in Table 5. It can be found that all the metrics performed better except for the specificity on the cross-validation set, which was slightly weaker than 2D CNN, and the method had an AUROC value of 0.9671 under the independent set, which further proves that the algorithm in this paper has a high generalization capability. The main reason for this result is that the original paper used more positive samples for training the model, with fewer positive examples remaining to evaluate the applicability of the model. However, a set partitioning ratio of 9:1 (cross validation dataset: independent dataset) was applied in this experiment, and although this may lead to some performance loss, the best results obtained in the independent dataset were still good: sensitivity of 66.67%, specificity of 93.63%, accuracy of 91.33%, and MCC of 0.528.

**TABLE 5:** Comparison with the experimental results of 2D CNN in the same setting.

Classifier	Cross-validation				Independent			
	Sens	Spec	Acc	MCC	Sens	Spec	Acc	MCC
2D CNN (Tao et al., 2020)	76.6	93.5	89.7	0.7	65.8	90.3	87.9	0.46
This Methods	98.168	90.736	94.718	0.8974	81.58	94.84	93.54	0.6839

## Conclusion

In this paper, we used the SMOTE algorithm with different parameters to address the sample imbalance of the dataset. The results show that this strategy can obtain a better result in terms of managing sample imbalance. In this process, ASDC as the feature extraction algorithm and LightGBM as the classification algorithm by comparing the results of various algorithms and descriptors. The combination obtained the best performance, and compared to other advanced neural networks, it achieved a significant improvement in all the typical measurement indexes. Under the same experimental setup, the method in this paper improves the accuracy by 5.64% in the independent test set and 0.2239 in the MCC metric relative to 2D-CNN. For the future research, graph neural networks (Zeng et al., 2020; Chen Y et al., 2021) and unsupervised learning (Xu et al., 2019a; Xu et al., 2019b) can be considered for performance improvement.

## Data Availability

Publicly available datasets were analyzed in this study. This data can be found here: https://github.com/khanhlee/snare-cnn.
